# Compound heterozygous missense mutations in a Chinese mucopolysaccharidosis type VI patient: a case report

**DOI:** 10.1186/s12886-021-01979-3

**Published:** 2021-05-13

**Authors:** Ming-Fang He, Ji Yang, Meng-Jie Dong, Yin-Ting Wang, Hai Liu

**Affiliations:** 1 Department of Ophthalmology, Affiliated Hospital of Yunnan University, Second People’s Hospital of Yunnan Province, Kunming, China; 2 The Eye Disease Clinical Medical Research Center of Yunnan Province, Kunming, China; 3 The Eye Disease Clinical Medical Center of Yunnan Province, Kunming, 650000 China

**Keywords:** Mucopolysaccharidosis type VI, *ARSB* gene missense mutation, Corneal opacity

## Abstract

**Background:**

Mucopolysaccharidosis type VI (MPS VI) is a rare autosomal recessive inherited disease caused by mutations in the arylsulfatase B (*ARSB*) gene. MPS VI is a multisystemic disease resulting from a deficiency in arylsulfatase B causing an accumulation of glycosaminoglycans in the tissues and organs of the body.

In this report, we present the case of a 16-year-old Chinese male who presented with vision loss caused by corneal opacity. MPS VI was confirmed by genetic diagnosis.

**Case presentation:**

A 16-year-old Chinese male presented with a one-year history of binocular vision loss. The best-corrected visual acuity was 0.25 in the right eye and 0.5 in the left eye. Although slit-lamp examination revealed corneal opacification in both eyes, the ocular examinations of his parents were normal. At the same time, the patient presented with kyphotic deformity, short stature, joint and skeletal malformation, thick lips, long fingers, and coarse facial features. Genetic assessments revealed that *ARSB* was the causative gene. Compound heterozygous missense mutations were found in the *ARSB* gene, namely c.1325G > A (p. Thr442Met) (M1) and c.1197G > C (p. Phe399Leu) (M2). Genetic diagnosis confirmed that the patient had MPS VI.

**Conclusions:**

This paper reports a case of MPS VI confirmed by genetic diagnosis.

MPS VI is a multisystem metabolic disease, with corneal opacity as a concomitant ocular symptom. As it is difficult for ophthalmologists to definitively diagnose MPS VI, genetic testing is useful for disease confirmation.

**Supplementary Information:**

The online version contains supplementary material available at 10.1186/s12886-021-01979-3.

## Background

Mucopolysaccharidosis type VI (MPS VI) is a rare lysosomal storage disease caused by deficient activity of arylsulfatase B (ARSB) [1]. The *ARSB* gene encodes the lysosomal enzyme ARSB, also known as N-acetylgalactosamine 4-sulfatase. This gene (NM_000046.5) is located on chromosome 5q13-q14 and contains eight exons and seven introns encoding 533 amino acids [2]. The abnormal accumulation of glycosaminoglycans (GAGs), dermatan sulfate, and chondroitin 4-sulfate resulting from a deficiency of ARSB could lead to MPS VI, which involves multiple organ systems and displays genetic and phenotypic heterogeneity [3]. This disease has various clinical manifestations, including short stature, corneal opacity, cardiac abnormalities, coarse facial features, hepatosplenomegaly, multifunctional disorders, joint stiffness, spinal protrusion, thick lips, and long fingers [4]. Here, we report the case of a 16-year-old patient who presented with vision loss; clinical examination revealed that the patient had corneal opacity and physical deformities. MPS VI was ultimately diagnosed by genetic analysis.

## Case presentation

**Fig. 1:**
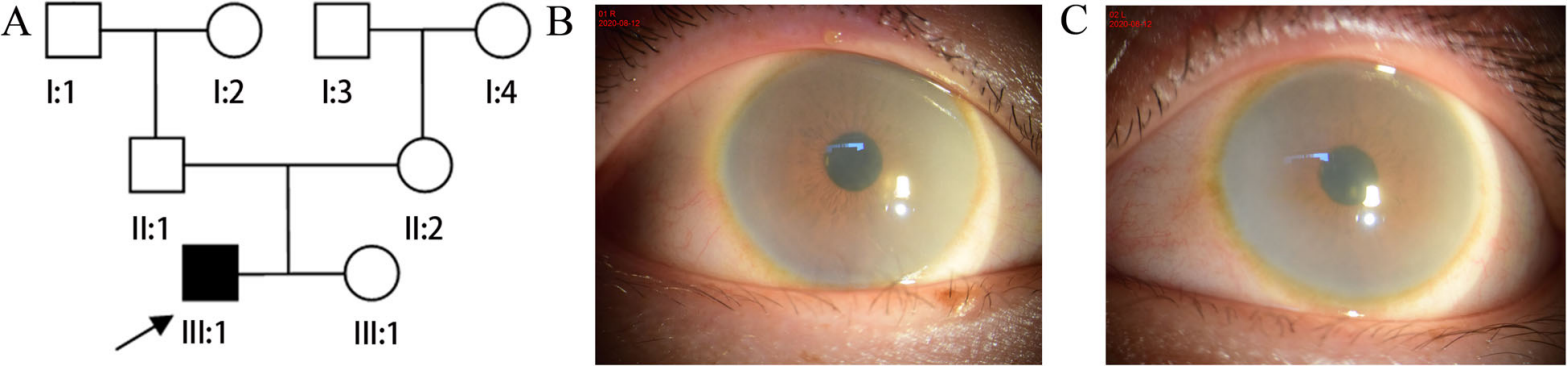
Pedigree and clinical examination of the proband. **a** Males are denoted with squares and females with circles. Empty and filled symbols indicate unaffected and affected individuals, respectively. Arrow represents the proband. **b**, **c** Anterior segment photographs of the (**b**) right and (**c**) left eye of the proband show corneal opacification in both eyes

A 16-year-old male patient visited our ophthalmology department due to deteriorating vision in both eyes over the past year. His parents were not related to one another, he was delivered by cesarean section after 38 weeks and weighed 2600 g at birth, and his parents and sister presented with normal vision and stature (Fig. 1a). His parents said the patient was normal at birth, but developed skeletal abnormalities at age one. When he was 9 years old, the patient visited the Beijing Children’s Hospital, but there was no definitive diagnosis. Slit-lamp examination of both eyes demonstrated corneal haze (Figs. 1b and c), and a Goldmann applanation tonometer showed that his intraocular pressure was 26.7 mmHg and 17.3 mmHg in his right and left eye, respectively. Uncorrected visual acuity was 0.25 in his right eye and 0.5 in his left eye. Of note, his best-corrected visual acuity did not increase: right eye + 4.5 DS/− 1.5 DC = 0.25, left eye + 3.5 DS/− 2.0 DC = 0.5. The axial length measured using an ultrasonic pachymeter showed the right eye to be 20.39 mm and the left eye to be 20.27 mm. Endothelial cell counts could not be determined due to the corneal opacity of the patient. Although the patient exhibited normal intelligence, physical examination revealed kyphotic deformities, short stature (compared to peers), skeletal and joint deformity, sternum herniation, thick lips, long fingers, coarse facial features, and a flat nasal bridge. Taken together, we could not provide a definite diagnosis according to the ocular manifestations with systemic manifestations, but we suspected that he had a rare hereditary syndrome. Therefore, we recommend the consanguineous family to make genetic diagnosis. All subjects provided signed informed consent, this study was approved by the Ethics Committee of the Affiliated Hospital of Yunnan University and was performed in accordance with the Declaration of Helsinki. Peripheral venous blood samples (5 ml) were drawn from the patient and his parents for genetic testing.

**Fig. 2:**
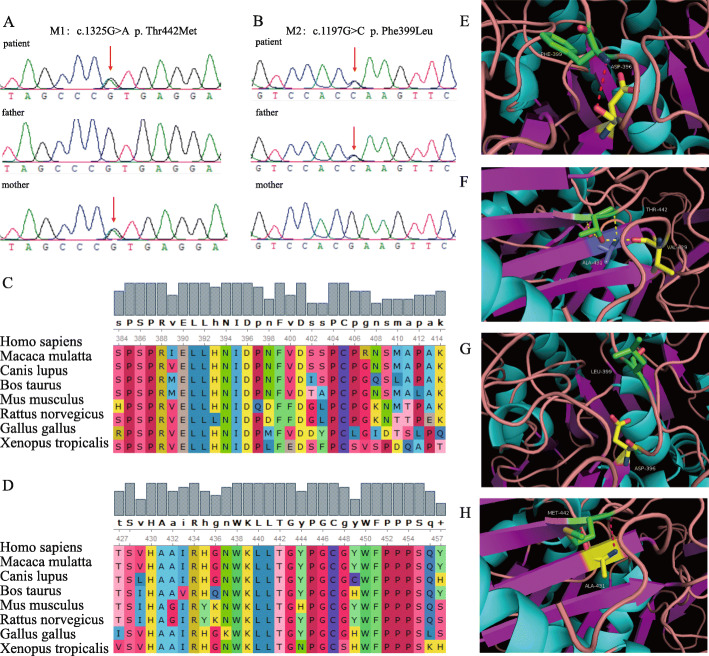
Mutation analysis of *ARSB*. **a**, **b** There were two mutations in the *ARSB* gene, i.e. c.1325G > A (p.Thr442Met) (M1) and c.1197G > C (p.Phe399Leu) (M2). Sequencing results showed that c.1325G > A (p. Thr442Met) was inherited from the mother, whereas c.1197G > C (p. Phe399Leu) was inherited from the father. **c**, **d** Mutant amino acids (No. 399, phenylalanine and No. 442, threonine) are highly conserved across species. **e**, **f**, **g**, **h** Molecular modeling of the (**e**, **f**) wild-type and (**g**, **h**) mutant ARSB protein. Structural modeling of the ARSB protein demonstrated that mutation of amino acid No. 399 (phenylalanine) resulted in the breakage of hydrogen bonds, and mutation of amino acid No. 442 (threonine) resulted in the partial breakage of hydrogen bonds

Genetic assessments revealed two heterozygous missense mutations in the *ARSB* gene, exon 7 c.1325G > A (p.Thr442Met) (M1) and exon 6 c.1197G > C (p.Phe399Leu) (M2), co-segregated with the disease phenotype in this family, and his healthy parents were heterozygous carriers (father: c.1197G > C, mother: c.1325G > A) (Figs. 2a and b).

The mutated areas were found in a highly conserved segment of the ARSB protein in humans and other species, and affected amino acids 399 (phenylalanine) and 442 (threonine) (Figs. 2c and d). According to the American College of Medical Genetics and Genomics, and analysis of four bioinformatics platforms (SIFT, Mutation Taster, Polyphen2, and REVEL), M1 was proposed as a pathogenic mutation and M2 was classified as likely pathogenic [5]. The global MAF of p.Phe399Leu was low [MAF(gnomAD) = 0.003]; however, the global MAF of p.Thr442Met was not found in the databases. Structural modeling of the ARSB protein (Figs. 2e and f) demonstrated that both Phe399 and Thr442 were located in the first β-sheet, which was required for receptor binding. The mutation caused the hydrogen bond between the amino acids to break (Figs. 2g and h), which resulted in protein structural instability. The patient was confirmed to have MPS VI because of the compound heterozygous mutations of *ARSB*.

## Discussion

Approximately 163 mutations of *ARSB* have been reported to date, among which missense and nonsense mutations are the most common [6]. In concordance with the mutation of *ARSB* and the lack of ARSB activity, GAGs cannot be completely degraded in lysosomes, which leads to the deposition of its substrates, further resulting in MPS VI [7]. Patients with MPS VI show light or heavy development with a varying age of onset and disease progression. The early diagnosis of patients with lightly-developed MPS is challenging, as they have a slow progression and no typical clinical features of MPS VI in the early stages of the disease [8]. Heavy MPS VI typically has an age of onset before 2 or 3 years, and patients usually present with short stature, scoliosis, cardiopulmonary dysfunction, cervical spinal cord compression, coarse facial features, and normal intelligence; these patients typically die from cardiopulmonary complications between the ages of 20 and 30 years [9]. Swiedler et al. presented a multicenter and multinational study of up to 123 patients, and suggested that the rate of disease progression in MPS VI could be determined based on urinary glycosaminoglycan (uGAG) levels. When the uGAG to creatinine ratio was > 100 μg/mg, such patients exhibited rapid disease progression and died before the age of 20 years. If the uGAG to creatinine ratio was < 100 μg/mg, these patients had a longer survival time [10]. MPS VI diagnosis can be confirmed by detecting ARSB activity in peripheral blood leukocytes or fibroblasts, or through *ARSB* gene testing, which not only facilitates further diagnosis, but also helps to elucidate the relationship between genotype and phenotype [6]. Compound heterozygous mutations exon 7 c.1325G > A (p.Thr442Met) and exon 6 c.1197G > C (p.Phe399Leu) in *ARSB* causing MPS VI have been previously reported, with Chupong et al. presenting data on four MPS VI patients, one of patient exhibited novel compound heterozygous missense *ARSB* mutations [11]. In the present case, we report the case of a 16-year-old male patient with MPS VI, which was confirmed by genetic testing. Both patient and his parents were genetically tested in this study, and two mutations were identified in the *ARSB* gene, namely c.1325G > A (p.Thr442Met) (M1) and c.1197G > C (p.Phe399Leu) (M2). The mutations inherited by the proband from his healthy parents were confirmed by family separation verification. Corneal opacity is a common clinical symptom of MPS VI that can present early on in the disease; such patients can develop other ocular complications, including glaucoma and optic neuropathy [12]. Thus, clinical ophthalmologists should possess increased disease awareness. Moreover, ophthalmologists should adequately assess the general condition of patients and note the family history of hereditary diseases when assessing corneal turbidity in patients who display concomitant growth and development disorders and skeletal abnormalities. If necessary, genetic testing should be undertaken to avoid misdiagnosis and missed diagnosis [13]. Overall, the present report details the confirmed diagnosis of MPS VI through genetic testing in a patient who presented with vision loss.

## Supplementary Information

**Additional file 1.**

## Data Availability

All data generated and/or analysed during this study are included in this published article and sanger sequencing results are in additional supporting files.

## Abbreviations

MPS VI: Mucopolysaccharidosis type VI
ARSB: Arylsulfatase B
